# Assessment of differentially expressed genes from in vitro matured human oocytes: A bioinformatics approach

**DOI:** 10.5935/1518-0557.20240030

**Published:** 2024

**Authors:** Gabriel Acácio de Moura, Mayara Lobato Lourenço, Yasmim Mendes Rocha, João Pedro Viana Rodrigues, Cristian Vicson Pinheiro, Alice Soares de Queiroz, Eduardo de Paula Miranda, Sebastião Evangelista Torquato Filho, Roberto Nicolete

**Affiliations:** 1 Postgraduate Program in Pharmaceutical Sciences (PPGCF), Federal University of Ceará (UFC), Fortaleza - Ceará, Brazil; 2 Oswaldo Cruz Foundation - Fiocruz Ceará, Eusébio - Ceará, Brazil; 3 Evangelista Torquato Human Reproduction Clinic, Fortaleza - Ceará, Brazil; 4 Postgraduate degree in natural resources biotechnology, Federal University of Ceará (UFC), Fortaleza - Ceará, Brazil

**Keywords:** fertility, reproductive techniques, oocyte, biology, gene, computational biology

## Abstract

**Objective:**

One of the techniques that has gained much attention is the in vitro maturation of oocytes for patients who use assisted reproduction techniques. However, its results are still inferior to controlled ovarian stimulation methodologies. Understanding the maturation mechanisms based on analyses can help improve this methodology’s results. The work aims to identify the central genes differentially expressed in oocytes after in vitro maturation in the germinal vesicle and metaphase II stages.

**Methods:**

This work is a computational analysis. The entire search will be conducted using the Gene Expression Omnibus (GEO) database. To carry out and obtain the data present in the work, an advanced research search was carried out in the GEO database within the period from January 1, 2013, to January 1, 2023. A total of 27 genomic data were available in the GEO database, of which only two were used.

**Results:**

Two datasets were identified on the Gene Expression Omnibus database platform: registration data GSE158802 and GSE95477. From the analysis, we identified five downregulated and thirty-six upregulated genes; the central genes that correlated with the main gene proteins found were CLTA and PANK1.

**Conclusions:**

There was a differential regulation of gene expression. The most central ones are related to energy capture.

## INTRODUCTION

In the current context, an increase in maternal age at first pregnancy can be observed, mainly due to the evolution of social and cultural changes such as educational level, financial establishment, and marital stability (Correa-de-Araujo & Yoon, 2021). This postponement leads to progressive losses of ovarian follicles, a decline in oocyte quality, and aneuploidy during the oocyte maturation stages, which leads to future natural sterility (Cao *etal.,* 2020). One of the ways to prevent these sterility conditions is the cryopreservation of matured oocytes or even oocyte in vitro maturation (IVM) technology. This last technique allows specialists to access oocytes with minimal stimulation on any day of the menstrual cycle. However, the clinical results of IVM are still inferior to controlled ovarian stimulation programs (Kirillova *et al.,* 2021).

*In vivo,* oocytes begin meiosis and stop at the diplotene stage of prophase I, morphologically presenting a characteristic nucleus called the germinal vesicle (GV), which remains quiescent for several years until the woman enters the reproductive life, with the hormone luteinizing (HL) response resumes meiosis reaching the metaphase II (MII) nuclear maturation stage (Llonch *et al.,* 2021). In IVM, this entire process is simulated in vitro to guarantee the competence of the oocyte for subsequent fertilization, which may be associated with the ability to resume and complete nuclear maturation with the correct formation of the meiotic spindle, undergo cytoplasmic maturation and support the embryonic development after fertilization (Xu & Zelinski, 2022).

It is known that the majority of oocytes recovered for in vitro fertilization (IVF) procedures do not have the developmental competence to obtain a viable blastocyst, and understanding how oocytes acquire this competence can define the reproductive success of the technique for these patients (Arroyo *et al.,* 2020). One way to predict the success of maturational quality is from the gene expression of these structures that can qualitatively and quantitatively indicate the transcriptional profile of the gamete (Hu *et al.,* 2022). Therefore, the present work aims to identify the central genes differentially expressed in oocytes after in vitro maturation at GV and MII stages.

## MATERIALS AND METHODS

### Type of Study

This work is a computational analysis; therefore, it is unnecessary to present it to the Ethics and Research Committee with human beings since the data included in the research are available in publicly accessible databases. The entire search will be conducted using the Gene Expression Omnibus (GEO) database.

## Data Search

To carry out and obtain the data present in the work, an advanced research search was carried out in the GEO database within the period from January 1, 2013, to January 1, 2023. The descriptors used were “oocyte maturation”, “maternal age ” and “human”. Data extracted from humans were used as inclusion and exclusion criteria: duplicate data, data that did not address the proposed theme, and data extracted from animals were removed.

**Figure 1:**
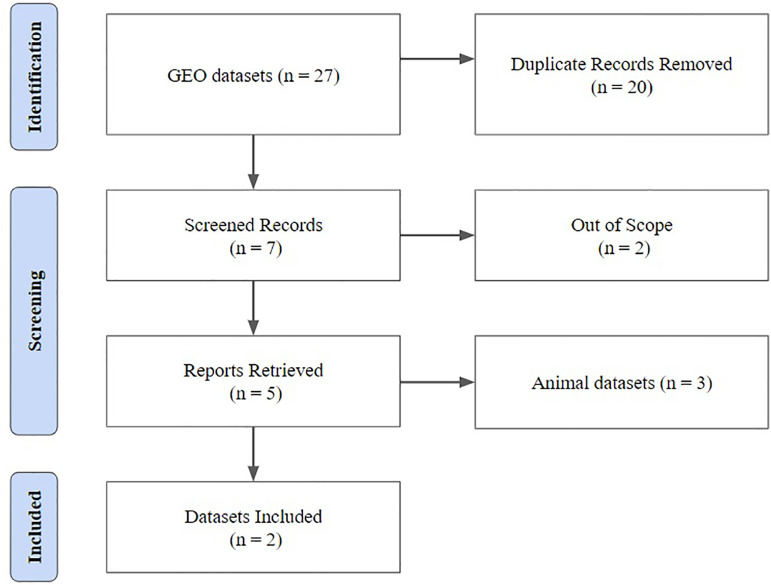
Methodological Flowchart for Obtaining Gene Expression Data.

A total of 27 genomic data were available in the GEO database, of which only two were used according to the inclusion and exclusion criteria discussed previously. All data screening is public in Figure 1. At the end of the search stage, we selected the data set with registration numbers GSE158802 and GSE95477. Information about the data set is available in Table 1.

**Table 1:** Extracted Dataset for Results Evaluation.

GEO Datasets	GSE158802	GSE95477
**Year**	2021	2018
**Country**	Spain	USA
**Platform**	GPL16791	GPL20301
**Number of oocytes**	75	20
**Group (GV)**	44	10
**Group (MII)**	31	10

Caption: GEO, Gene Expression Omnibus.

### Differentially Expressed Genes Screening

To carry out the gene screening, we used a methodology based on the article by Wu *et al.,* (2022). The different results were obtained using the online data analysis tool GEO2R (software) to compare gene expression between the groups. Differentially expressed genes (DEG) were selected, and the accepted screening standard was *p*<0.05 and [Log 2 FC] > 2.0. The genes with the top 25 expression rates were chosen for further analysis for a volcano plot (volcano-shaped graph) and also a heat map, which expressively shows the genes that were most expressed within certain groups using a color scale generated from the R Studio Software (Version 4.2.3).

### Gene Enrichment Analysis

We used a methodology adapted from Wang *et al.* (2021a) for genetic enrichment analysis. To this end, after selecting the 23 genes that obtained significant expressions from the GSE158802 and GSE95477 datasets, they were transferred to the inbox of the Metascape Web Server, which works as an annotation and visualization tool in databases. Kyoto Encyclopedia of genes and Genomes (KEGG) and Gene Expression Omnibus (GEO) database. The data extracted from the analysis were a list of biological processes, an enrichment list of cellular signatures, and data relating to protein-protein interaction (IPP).

## RESULTS

### Differential Identification of Genes Between Groups

During the search on the platform, both data sets made groups available in two stages: GV and MII. However, only one of them was stratification by age, so we conducted a differential analysis of genes without considering the maternal age factor. The number and group of patients available for the study are shown in Table 1.

**Figure 2:**
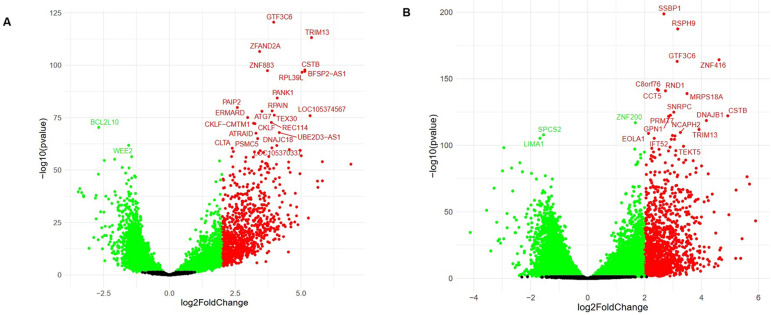
Volcano Plot of Differential Gene Expression in the Analyzed Datasets. Caption: It was observed the differential expression between GV and MII groups. In A, the magnitude of change in the GSE158802 dataset. The importance of the transition from the GSE95477 data set can be observed in B. All data was generated from the GEO2R web server. In red, the positively and in green, negatively regulated genes for the cutoff point *p*<0.05 and [log 2 FC] > 2.0.

**Figure 3:**
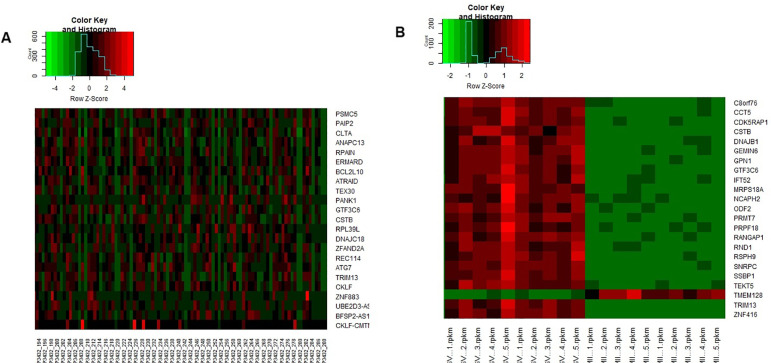
Heatmap and Expression of the Top 23 Genes in the Datasets. Caption: The expression of the top 23 genes in each dataset can be observed. The presentation by heat map in the GSE158802 dataset can be followed in A. In B, the expression of genes in the GSE95477 set can be observed. In red, positively regulated genes can be marked; in green, negatively regulated genes. The cutoff point adopted was *p*<0.05 and [log 2 FC] > 2.0. R Studio Software generated graphics.

The analysis using the GEO2R database identified 8839 genes, of which 3437 were expressed in common between both groups in the GSE158802 dataset. In the analyzed data set GSE95477, a total of 16046 genes were identified, and of these, 5979 were expressed in common between both groups. Furthermore, negatively and positively regulated genes expressed in oocytes were verified, as shown in Figures 2 and 3.

### Gene Enrichment Analysis

After analyzing the identification of genes differentially expressed in GV and MII oocytes, we identified five negatively and 36 positively regulated genes. After verification, these underwent gene enrichment analysis using the Metascape Web Server. Data relating to the genes used in the study are available in Table 2.

**Table 2:** Differentially Expressed Genes Selected for Gene Enrichment Analysis.

Group	Total	Genes
Downregulated genes	05	*BCL2L10, WEE2, LIMA1, SPCS2, ZNF200*
Upregulated genes	36	GTF3C6, TRIM13, ZFAND2A, ZNF883, CSTB, BSFP2-AS1, RPL39L, PANK1, PAIP2, RPAIN, ERMARD, ATG7, TEX30, CKLF-CMTM1, CKLF, REC114, ATRAID, DNAJC18, UBE2D3-AS1, CLTA, PSMC5, LOC105370337, SSBP1, RSPH9, ZNF416, C8orf76, RND1, CCT5, MRPS18A, SNRPC, CSTB, PRMT7, GPN1, EOLA1, IFT52, TEKT5

The process enrichment analysis carried out from the genes obtained verified six main biological functions that the clusters could be linked to: chaperone-mediated protein folding, regulation of protein catabolic processes, involvement in peptide metabolic processes, axon guidance, biogenesis and mutation of organelles, and formation of the set of cilia. Data is available in Table 3.

**Table 3:** Top Genetic Enrichment Clusters for Biological Processes.

Number of GO	Category	Description	Number of Genes	%	Log10(p)	Log10(q)
GO:0042176	GO Biological Process	Regulation of Protein Catabolic Processes	04	10.53	−2.96	0.00
GO:0006518	GO Biological Process	Peptide Metabolic Process	04	10.53	−2.42	0.00
R-HSA-422475	Reactome Gene Pool	Axon Guidance	04	10.26	−2.30	0.00
R-HSA-1852241	Reactome Gene Pool	Organelle Biogenesis and Maintenance	03	7.89	−2.22	0.00
GO:0060271	GO Biological Process	Eyelash Set	03	7.89	−2.10	0.00
GO:0061077	GO Biological Process	Establishing the Localization of Proteins in the Organelle	03	7.89	−2.02	0.00

Caption: Number of Genes: number of genes provided by the user; %: percentage of genes provided by the user; Log10(p): p-value in base 10 log; Log10(q): log-adjusted *p*-value of base 10. *p*-value < 0.01, minimum count of 3, and enrichment factor >1.5 (observed counts/expected counts). Data obtained by the Metascape Web Server.

**Figure 4:**
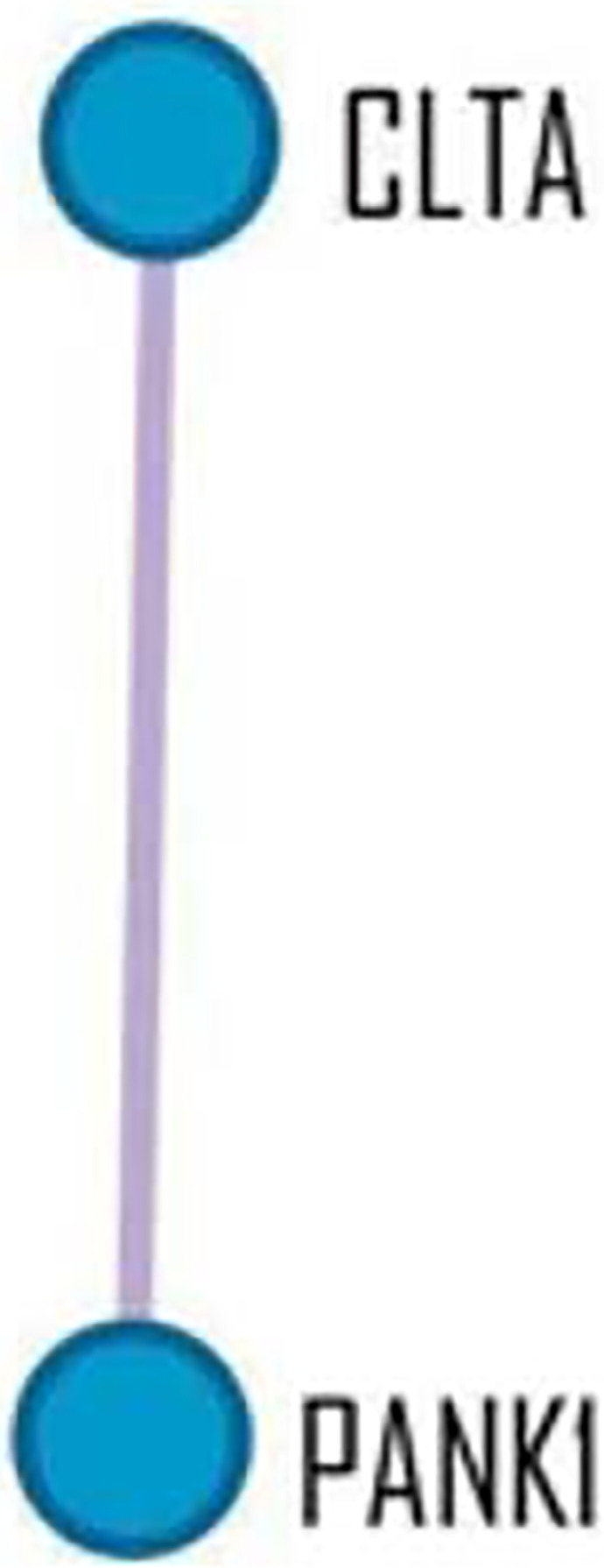
MCODE Protein-Protein Interaction Network. Caption: The Metascape library performed a protein-protein interaction enrichment analysis for each selected gene in the databases. Those with STRING physical scores > 0.132 were used. The resulting network contains a subset of proteins that form physical interactions with at least one list member.

Furthermore, we analyzed their cellular signature to characterize the genes of interest further. This made it possible to identify three main clusters, as shown in Table 4. As expected, two main groups of ovarian cells were identified; however, what caught our attention was the identification of cellular signatures for other lineages of interest in the case of cells responsible for the skeletal muscles. After identifying the initial genes and analyzing their biological functions, we checked the main gene proteins that interact with the other genes found. This analysis verified two central genes, namely the *CLTA* and *PANK1* genes. Data available in Figure 4.

**Table 4:** Summary of Enrichment Analysis for Different Cellular Signatures.

GO ID.	Description	Number of Genes	%	Log10(p)	Log10(q)
M41745	Rubenstein Skeletal Muscle FAP Cells	03	7.90	−2.80	0
M41712	Ovarian Fan CL10 Putative Granulosa Cell Early Atresia	03	7.90	−2.30	0
M41710	Ovarian Fan CL08 Mature Cumulus Granulosa Cells 2	04	11.00	−2.10	0

Caption: Number of Genes: number of genes provided by the user; %: percentage of genes provided by the user; Log10(p): *p*-value in base 10 log; Log10(q): log-adjusted *p*-value of base 10. *p*-value < 0.01, minimum count of 3, and enrichment factor >1.5 (observed counts/expected counts). Data obtained by the Metascape Web Server.

## DISCUSSION

Currently, in assisted reproduction techniques (ART), around 60 to 70% of women cannot get pregnant, and several factors can cause such implantation failure (Benkhalifa *et al.,* 2022). Among these factors is the quality of adequately matured oocytes, which is decisive for fertilization and the embryo’s adequate development in ART (Nevoral *et al.,* 2016). IVM of oocytes is already becoming a reality and is no longer just an experimental technique that aims to assist in ART effectively (Kirillova *et al.,* 2021). Therefore, understanding the mechanisms of maturational success and failure during IVM can help to significantly improve its clinical outcomes (Ruebel *et al.,* 2021).

Oocyte maturation is continuously monitored to allow practical expression of genes highly dependent on post-transcriptional regulation of messenger RNA (mRNA). Therefore, genomic annotations can be used to understand the maturational bioprocesses of *in vitro* oocytes (Lin *et al.,* 2022). GEO database is the largest and most comprehensive public genetic and gene expression database and, therefore, has been continuously used in exploratory basic bioinformatics research (Wu *et al.,* 2022). This research model allows the screening and identification of DEGs among different groups. In addition, these annotations can serve as a basis for identifying genetic hubs and assembling IPPs (Guo *et al.,* 2019).

The data generated from GEO2R identified 24,885 genes in both data sets, which shows the complexity that maturational processes may require during maturation. This is due to the activation of molecular pathways underlying transcription from oocyte to embryo, which is highly dependent on maternal RNAs and proteins accumulated during oocyte growth in oocyte maturation (Elis *et al.,* 2008). This would also explain the elevation and reduction of gene expression between the different groups in the GV and MII stages. In addition, our enrichment analysis shows that the hubs identified from the 25 most expressed genes are primarily associated with biological processes.

Protein folding mediated by chaperones was one of the most significant processes across the selected genes. Chaperones are a family of proteins that ensure protein homeostasis, mediating folding, trafficking, sequestration, and renewal of cellular proteins, of which in the human genome, more than 300 genes encode these structures (Braun, 2023). Previous studies have already highlighted the importance of chaperones in the process of oocyte competence. The study by Smith *et al.* (2022) found that the H3.3 chaperone Hira complex is intrinsically linked with oocyte quality. In another study, the histone/chaperone complexes Asf1a and Asf1b were considered necessary for embryo implantation, demonstrating how much these genes can impact the outcome of fertilization processes (Wang *et al.,* 2021b).

The regulation of protein catabolic processes was another biological function found alongside the biogenesis and regulation of organelles. Both methods may be intrinsically linked. In a previous article published by Rodríguez-Nuevo *et al.* (2022) it was found that *Xenopus oocytes* avoid reactive oxygen species by remodeling the mitochondrial electron transport chain by eliminating complex I. In another study carried out by Ma *et al.* (2016), in mice with Rab protein knockdown, there was a dysregulation in the endoplasmic reticulum and, as a consequence, in its physiological processes, which confirms our hypothesis that protein catabolic processes may be associated with the regulation of organelles.

In the literature, Jia & Wang (2020) tested the effectiveness of C natriuretic peptide on the competence of bovine oocytes matured in vitro, and it was found that there was an improvement in the oocyte development potential of oocytes from large follicles of approximately (3-8 mm). Furthermore, there is already an association that some peptides, such as growth hormone, can assist in oocyte maturation, leading to better morphological results in embryo development and cleavage rate (Chang *et al.,* 2022). This result agrees with the data generated by the Metascape Web Server, which indicates that one of the main clusters of genes obtained precisely regulated the peptide metabolic process.

Two hubs found according to the selected genes were axon guidance and a set of cilia, which may be correlated with the expression of the other selected genes. However, no correlations were found in the literature that could demonstrate such findings during in vitro maturation processes. On the other hand, something that may explain the enrichment analysis results is that the selected genes are not expressed only in oocyte cells, as demonstrated in the cellular signatures that identified that the selected genes could be expressed in different cells.

It also checked the IPP to investigate the central interacting genes of those that were selected. An important connected hub visualized during the IPP analysis was the interaction between the *CLTA* and *PANK1* genes. *CLTA* is a gene that encodes the clathrin protein that acts directly in the coordinated and complex uptake of the clathrin coat at the plasma membrane, which is then internalized as a vesicle and endocytoses molecules via a receptor (Grimm *et al.,* 2022). The *PANK1* gene encodes members of the pantothenate kinase family responsible for the general control of the biosynthesis of coenzyme A, which was recently identified as a mediator of gluconeogenesis (Yang *et al.,* 2020). This interaction points to mechanisms for obtaining molecules and energy for proper oocyte development.

The study’s main limitations are that patients with reproductive complications were used in one of the data sets, which could bias evaluations based on unhealthy patients. Furthermore, the lack of information regarding the age of each donor in one of the data sets limited the findings of the present work.

Therefore, we concluded that there was a differential expression of core genes in MII stage oocytes compared to GV. Among these primary genes, those that maintained a central interaction with all those identified were the CTLA and PANK1 genes responsible for maintaining these gametes and obtaining energy through gluconeogenesis. Our analyzes stand out for identifying genes that can be used in future research to better understand gamete development.
